# An East Antarctic, sub-annual resolution water isotope record from the Mount Brown South Ice core

**DOI:** 10.1038/s41597-024-03751-w

**Published:** 2024-09-10

**Authors:** Vasileios Gkinis, Sarah Jackson, Nerilie J. Abram, Christopher Plummer, Thomas Blunier, Margaret Harlan, Helle Astrid Kjær, Andrew D. Moy, Kerttu Maria Peensoo, Thea Quistgaard, Anders Svensson, Tessa R. Vance

**Affiliations:** 1 Physics of Ice Climate and Earth, Niels Bohr Institute, University of Copenhagen, Tagensvej 16, 2200 Copenhagen, Denmark; 2 Research School of Earth Sciences, Australian National University, Building 142, Mills Road, Acton, ACT 2601 Australia; 3 Australian Centre for Excellence in Antarctic Science, Australian National University, Canberra, ACT 2601 Australia; 4 ARC Centre of Excellence for Climate Extremes, Australian National University, Canberra, ACT 2601 Australia; 5 Climate and Environmental Physics, Physics Institute and Oeschger Centre for Climate Change Research, University of Bern, CH-3012 Bern, Switzerland; 6 Australian Antarctic Program Partnership, Institute for Marine & Antarctic Studies, University of Tasmania, Hobart, TAS 7004 Australia; 7 Department of Climate Change, Energy, the Environment and Water, Australian Antarctic Division, Kingston, TAS 7050 Australia; 8 Present Address: Second Sun ApS, Carit Etlars Vej 6, kld, 1814 Frederiksberg, Denmark; 9 Present Address: Department of Environmental Science-Atmospheric modeling, Aarhus University, Frederiksborgvej 399, 4000 Roskilde, Denmark

**Keywords:** Palaeoclimate, Infrared spectroscopy, Cryospheric science

## Abstract

We report high resolution measurements of the stable water isotope ratios (*δ*^18^O, *δ*D) from the Mount Brown South ice core (MBS, 69.11^°^ S 86.31^°^ E). The record covers the period 873 - 2009 CE with sub-annual temporal resolution. Preliminary analyses of surface cores have shown the Mount Brown South site has relatively high annual snowfall accumulation (0.3 metres ice equivalent) with a seasonal bias toward lower snowfall during austral summer. Precipitation at the site is frequently related to intense, short term synoptic scale events from the mid-latitudes of the southern Indian Ocean. Higher snowfall regimes are associated with easterly winds, while lower snowfall regimes are associated with south-easterly winds. Isotope ratios are measured with Infra-Red Cavity Ring Down Spectroscopy, calibrated on the VSMOW/SLAP scale and reported on the MBS2023 time scale interpolated accordingly. We provide estimates for measurement precision and internal accuracy for *δ*^18^O and *δ*D.

## Background & Summary

With a plethora of paleoclimatic information contained in their continuous stratigraphy, Antarctic ice cores constitute unique archives of past climate variability. Deep cores drilled in the low accumulation areas of East Antarctica provide a picture of the climate spanning up to 800,000 y^1,2^, whereas higher accumulation areas can typically yield paleoclimatic signals of high temporal resolution (decadal to subannual)^3–5^. The isotopic composition of polar ice is a valuable paleoclimatic proxy of past hydrological cycle conditions and atmospheric temperatures^6–9^. It is commonly expressed with the *δ* notation^10^ as the deviation of a sample’s isotopic ratio relative to that of a reference water expressed in per mille (‰) as: *δ*^*i*^ = (^*i*^*R*_sample_/^*i*^*R*_V SMOW_ − 1) × 1000 where ^2^*R* = ^2^H/^1^H and ^18^*R* = ^18^O/^16^O. The internationally established reference scale is defined by two waters, the Vienna Mean Ocean Water (VSMOW) and the Standard Light Antarctic Precipitation (SLAP), both materials, stored, maintained and distributed by the International Atomic Energy Agency^11^.

**Table 1:** Instrument specifications for analyses at IMAS, Hobart and ANU, Canberra.

Component	IMAS, Hobart	ANU, Canberra
Picarro Model	Picarro L2130-*i*	Picarro L2140-*i*
Autosampler	Picarro A0325	Picarro A0325
Vaporizer	Picarro A0211	Picarro A0211
Syringe	Trajan SGE 002250 10 *μ*L	Trajan SGE 002250 10 *μ*L

In this work we present dual measurements (*δ*^18^O, *δ*D) from a new high-resolution ice core from East Antarctica, the Mount Brown South (MBS) ice core (69.11° S 86.31° E, 2084 m elevation). The MBS ice core was drilled during the 2017/2018 austral summer field season addressing a substantial need for millennial-length, high-resolution climate records from the Indian Ocean sector of East Antarctica^12^. The isotopic record presented here is a compilation of discrete analyses (4–104 m depth; 0.03 m resolution) and continuous flow analysis (CFA) (94 – 295 m depth; 0.005 m resolution). The record is 295 m long and represents 1135 years (873–2009 CE)^13^. We used state of the art Cavity Ring Down Spectroscopy (CRDS) assuring exceptional data quality with respect to both precision and accuracy together with quality metrics to quantify measurement noise as a function of depth. In particular, for the CFA part of the record we used innovative in-house developed methods with respect to ice core sample handling and flash, fractionation-free vaporisation^14^ that resulted in ultra-high resolution sub-annual isotope signals.

The MBS water isotope record will be useful for a wide range of studies aiming to understand the climate variability of the Southern Hemisphere spanning centennial to inter-annual time scales. Covering a time frame before, as well as during the observational window, it can be used for forcing climate models at high resolution. In particular, the location of the MBS drilling site could potentially allow for studies focusing on the dominant climate modes of the South Hemisphere, the El Niño Southern Oscillation (ENSO) and the Southern Annular Mode (SAM), as well as interdecadal variability stemming from the Pacific Ocean^12,13,15^.

A small part of the record (4-20 m of the *δ*^18^O signal) has previously been presented in the study of Jackson *et al*.^16^, which focuses on the recent climatology of the site and its implications for the interpretation of the *δ*^18^O signal in the core. An investigation of ENSO signals aparent in the MBS record has been performed in^15^ also using the top 20 m of the high resolution water isotope record.

## Methods

### The Mount Brown South (MBS) Ice Core

#### The ice core site

**Fig. 1:**
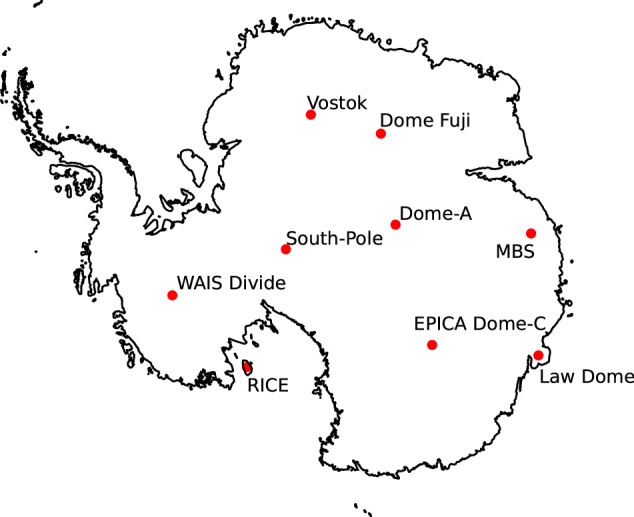
Map of Antarctica and location of prominent ice core drilling sites.

The MBS ice core site is located near the boundary of Princess Elisabeth and Wilhelm II Land in East Antarctica, 380 km inland and to the west of Australia’s Davis Station. It is located in the Indian Ocean sector of coastal East Antarctica, a region poorly represented by high-resolution ice core records, limited to several short cores (up to 350 years) in Princess Elizabeth Land^17^ and the Law Dome ice core^18^ (Fig. 1). The Law Dome record spans up to 90,000 years and is available at annual or sub-annual resolution for the past 2000 years. It has been widely used to reconstruct regional climate variability of coastal East Antarctica^19^ as well as hydroclimate variability in both southwest Western Australia^5,20,21^ and eastern Australia^22,23^ due to synoptic-scale weather and climate links between coastal East Antarctica and Australia^24^.

The MBS ice core site was selected following an extensive site-selection study^12^ to fill a critical gap in the current network of ice core records. Seven criteria were considered when selecting the ice core site including (i) at least 1000-year-old ice at depths of 300 m; (ii) summer surface temperatures <0°C to minimise melt; (iii) high-resolution (sub-annual) requiring accumulation of >250 mm a^−1^ (iv) minimal surface reworking; (v) located on a ridgeline or dome; (vi) teleconnection with mid-latitude climate; (vii) complementary to existing records. The final site, Mount Brown South, meets all of these requirements and expands the current network of high-resolution temperature and hydroclimate proxies. An annually resolved chronology for the MBS ice core, MBS2023, was developed in Vance *et al*.^13^ using annual layer counting and alignment to known volcanic horizons.

#### Drilling and sampling

**Fig. 2:**
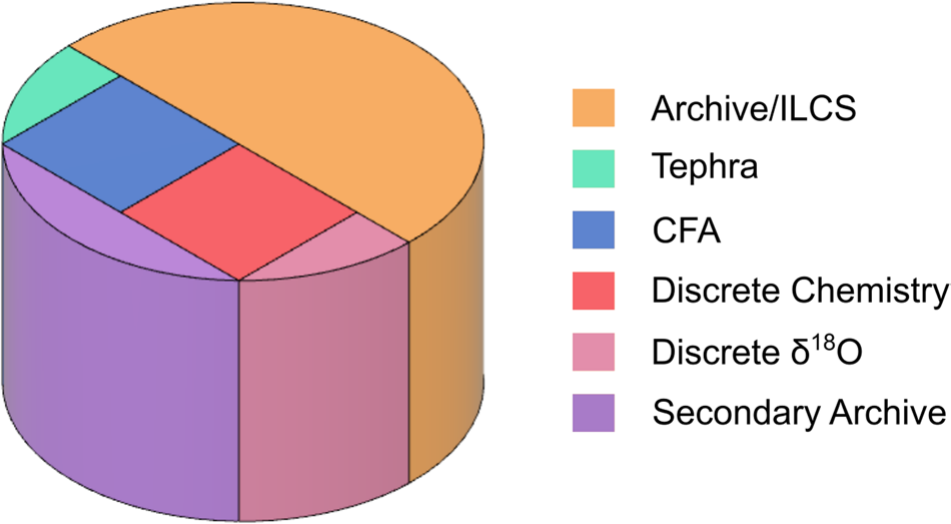
Cross section of the MBS ice core showing the cutting scheme of the ice core samples.

During the 2017/18 austral summer field season, a 295 m long ice core with a diameter of 98 mm was drilled from 4 m below the surface using a Hans Tausen drill^25^. Wet drilling started at a depth of 93 m (driller’s depth) using Estisol 140 as drilling liquid. The core was cut in 1 m sections (ice core bags) in the field and transported to the ice core facilities of the Institute for Marine and Antarctic studies (IMAS) in Hobart, Australia for further processing. Visual features in the ice such as breaks, wind crusts and bubble free layers were thoroughly logged prior to further sectioning. Following the cutting scheme shown in Fig. 2, samples were prepared at a temperature of −18°C using a trace-clean band saw. Two rectangular rods measuring 34 × 34 mm were cut for the purpose of CFA and for trace ion chemistry analysis. The two wedges on each side of the square rods were collected for tephra and discrete water stable isotope analysis. The latter was sampled at a resolution of 30 mm.

The CFA square rods were subsequently transported to Copenhagen, Denmark for further sample preparation and subsequent chemical and water isotope analysis. Typically, 1-2 mm from the ends of the CFA rods were carefully shaved off in order to minimise contamination of the chemical impurity analysis. For rods with slightly slanted ends the removal of material was adjusted and documented accordingly. For the same reason, we removed internal breaks and then shaved off and flattened the ice on each side of the break. The lengths of all the ice pieces were carefully documented, prior as well as after the processing of breaks and edges of the CFA rods.

### Stable Isotope Analysis – Cavity Ring Down Spectroscopy

Two versions of the Picarro water isotope analyzer (Picarro L-2130*i* and L-2140*i*) were used for the analysis of the discrete and continuous samples. The Picarro L-21*xxi* water isotopes analyzers are Cavity Ring Down Spectrometers (CRDS), operating in the near-IR region at 7200 cm^−1^ and both operate on the same spectroscopic principles. The analyzer uses a continuous emission diode laser source coupled to a high-finesse, 3-mirror, V-shaped cavity. Analysis takes place in the gas phase at a cavity pressure of 67 mbar (50 Torr) and a temperature of 80°C. Precise control of the cavity’s pressure and temperature (≈2mbar, 1mK, 1-*σ*) is key for optimal instrument performance. This is achieved by the instrument’s control software continuously adjusting the position of two proportional valves up- and downstream of the optical cavity maintaining stable pressure and by driving heat elements installed in a well insulated and ventilated box that contains the optical cavity. Temperature and pressure stability is also facilitated by maintaining a stable laboratory temperature and a stable gas flow at the inlet of the instrument. The sample flow through the optical cavity is $30\,{\rm{cc}}\,{\min }^{-1}$.

### Discrete samples water isotope analysis; 0-104 m depth

The upper 100 m of the ice core were analysed at the Australian Antarctic Division (AAD) Ice Core laboratory facilities at IMAS, Hobart (4.0–74.2 m) and the Australian National University (ANU), Canberra (74.2–100.0 m), for interlaboratory comparison purposes using a discrete sampling scheme. The discrete ice core samples were stored frozen at IMAS, Hobart after processing. For the section analysed at the ANU, Canberra, samples were transported frozen and remained frozen until analysis. The ice samples were melted at room temperature, whereafter 200 *μ*L were transferred into 2 mL screw thread glass vials (**Fisherbrand 033918**) fitted with 300 μl polyspring inserts (**Thermo Scientific C4010-S630**) and sealed with silicone/PTFE septum caps (**Thermo Scientific THC09-15-1178**). The ice samples at IMAS were defrosted in a fridge at 4^°^C overnight. Aliquots of 250 *μ*L were transferred into 2 mL snap top glass vials (**Agilent 5182-0546**) fitted with 250 *μ*L pulled point glass vial inserts (**Agilent 5183-2085**) and sealed with PTFE/red silicone snap caps (**Agilent 5182-0564**).

#### Instrumentation used for discrete samples

Stable water isotope analysis on the discrete samples was performed using two variants of the Picarro L21xx-*i* analyzer with similar performance characteristics with respect to precision and sample handling. At IMAS, Hobart, a Picarro L2130-*i* stable water isotope analyzer was used, while the L2140-*i* variant was used at ANU, Canberra. At both IMAS, Hobart, and ANU, Canberra, the Picarro analyzers were coupled to the Picarro A0211 vaporizer and the Picarro A0325 autosampler. Pressurized dry air ($\left[{{\rm{H}}}_{2}{\rm{O}}\right] < 2\,{\rm{ppmv}}$) was used as carrier gas and mixed with the injected sample in the vaporizer’s sample cell at a temperature of 110°C, regulated by means of PID control. The water sample was injected using a 10 *μ*L syringe (** SGE 10R-BT-GT-0.47C**) with an injection volume of 1.8 *μ*L liquid water at IMAS, Hobart and 1.5 μL at the ANU, Canberra. After an equilibration time of 60 s in the vaporizer’s cell, the vapor-air mixture was gradually introduced into the optical cavity for spectroscopic analysis. The instruments comprising each experimental setup as well as the specifications of the autosampler injection methods are given in Tables 1 and 2 respectively.

**Table 2:** Autosampler injection method for the Picarro L2130-*i* (analyses performed at IMAS, Hobart) and the Picarro L2140-*i* (analyses performed at ANU, Canberra).

Property	Picarro L2130-*i*	Picarro L2140-*i*
Syringe Volume	10 *μ*L	10 *μ*L
Sample Volume	1.8 *μ*L	1.5 *μ*L
Fill Speed	2.5 *μ*L/s	500 nL/s
Fill Strokes	1	1
Inject Speed	2.0 *μ*L/s	500 nL/s
Pre Injection Delay	0 s	7 0 s
Post Injection Delay	1.0 s	1.0 s

#### Run configuration for discrete analyses

Analysis of discrete samples in Hobart and Canberra followed similar protocols with small deviations. At both facilities, an analytical sequence consisted of calibration sequences bracketing “unknowns", with isotopic values expressed as per mil (‰) and relative to the Vienna Standard Mean Oceanic Water (VSMOW). A typical analytical sequence using the AAD Picarro L2130-i consisted of 30 “unknowns", while a sequence at the ANU, Canberra consisted of 20 “unknowns".

In Hobart, three in-house calibration standards were used (tap water, Dome Summit South (DSS) and Lambert Glacial Basin (LGB)), as well as a laboratory reference water sample or check standard, MBS, treated as an unknown (Table 3). The check standard (MBS) was analysed after 10 unknown samples to monitor run stability. Analysis was performed in “High-Precision" mode with methods that enabled analysis of a single injection in ~8 mins. A typical analytical run with calibration standards and 30 “unknowns" was completed in just over 24 hours. The analysis of each standard (including the check standard) consisted of 8 injections, with the first 4 injections discarded in order to account for memory effects. The “unknowns” were analysed sequentially with increasing depth to reduce the memory effects. Each “unknown” measurement consisted of 4 injections with the first injection discarded.

**Table 3:** ANU local standards used for the discrete analyses.

Standard	*δ*^18^O (‰)	*δ*D (‰)
**VSMOW**	**0**	**0**
**SLAP**	− **55.5**	− **427.5**
DSS	− 22.8	− 178.1
MBS	− 32.4	− 258.1
LGB	− 39.0	− 309.6

All values are given in ‰ with respect to VSMOW.

In Canberra, we used two in-house calibration standards (DSS and LGB), as well as a check standard, MBS, treated as an unknown. The check standard (MBS) was analysed between unknown samples #10 and #11 to monitor run stability, as well as at the mid-point of each calibration sequence. The analysis of each standard (including the check standard) consisted of 20 injections, with the first 13 injections discarded in order to account for memory effects. The “unknowns” were analysed sequentially with increasing depth to reduce the memory effects associated with large isotopic steps. Each “unknown” measurement consisted of 10 injections with the first four discarded. Analysis at the ANU, Canberra was performed in the Picarro “High-Precision” mode, which utilises injection times of ~9 min (total time ~1.5 hours per sample). A run consisted of several analytical sequences and thus lasted several days.

### Continuous Flow Analysis (CFA)

The more recent development of high-resolution Continuous Flow Analysis (CFA) techniques based on the continuous melting of ice core samples^26–29^ has yielded unprecedented records of chemical impurities^30–33^. We continuously melt an ice core rod over a custom designed and manufactured heated melt plate^29^ and further debubble and distribute the liquid sample stream to an array of analyzers. This approach has the potential to yield multi-component, very high resolution data sets on a common depth scale with a massively improved throughput compared to the laborious relatively low-resolution analysis performed in the traditional way using ion chromatography or inductively coupled plasma mass spectrometry.

Laser spectroscopy techniques employing Cavity Enhanced/Cavity Ring Down Spectroscopy (CRDS) configurations have made it possible to measure trace gas concentrations and stable water isotope ratios in a continuous flow mode with minimal sample preparation^34–36^. Interfacing these techniques to existing CFA systems allowed for continuous, high-resolution records of CH_4_^37,38^ as well as water stable isotope records (*δ*^18^O, *δ*D)^39–42^ in ice cores with significant improvements in precision, accuracy, sample consumption and throughput.

#### The Copenhagen CFA system

The Copenhagen CFA system is optimized for high depth resolution capable of analysing an array of chemical parameters such as conductivity, insoluble dust, Ca^2+^, ${\,\mathrm{NH}}_{4}^{+}$, Na^+^ and pH^29,32,43^. Following a modular approach, more components can be incorporated in the synchronous analysis. The system utilizes an aluminum melter with an outer sample square ring measuring 34 × 34 mm and an inner sample square ring measuring of 26 × 26 mm (Fig. 2 in^29^). The melt rate is controlled by the melter’s temperature maintained by four 125 W Mickenhagen heat cartridges in combination with a J-type thermocouple and a PID–loop control unit (**dTRON 308, Jumo**). A draw wire sensor incorporating an optical encoder (**WayCon SX-80**) records the melt speed with a sensitivity *γ* = 25 counts mm^−1^.

**Fig. 3:**
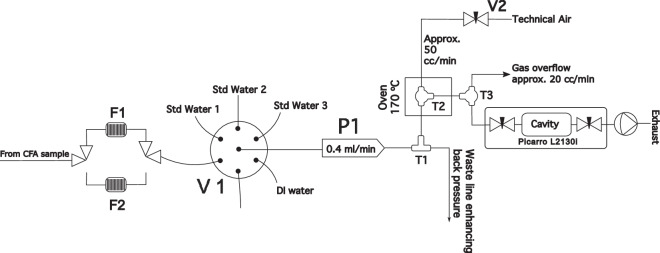
Block diagram of the water isotope analysis part of the CFA system. For a complete description of the system’s components, the reader is referred to Section Methods.

During the campaign the nominal melting speed of $3.5\,{\rm{cm}}\,{\min }^{-1}$ yielded a clean, central line sample flow of $24\,{\rm{ml}}\,{\min }^{-1}$ maintained by a peristaltic pump (**Ismatec IPC-16**). This sample contained ≈10% air due to the air bubbles occluded in the ice core. Removal of the air occurred in a homemade Polyether-Ether-Ketone (PEEK) flat triangular-cell debubbler following a simple buoyancy principle (Fig. 3 in^29^). Downstream of the debubbler, the air-free flow equalled $4\,{\rm{ml}}\,{\min }^{-1}$ of which $0.4\,{\rm{ml}}\,{\min }^{-1}$ was pumped further for water isotope analysis. The liquid water-air mixture with a flow of $20\,{\rm{ml}}\,{\min }^{-1}$ was forwarded to a gas extraction membrane (**3M, Liqui-Cel MM-0.75 × 1 Series**) for further gas-liquid separation and subsequent CH_4_ analysis. A more thorough presentation of the chemical analysis of the MBS core accompanied by the adjoined data records will follow in a separate publication. The dataset can be accessed at the Australian Antarctic Data Center^44^.

#### Continuous flash vaporisation for water isotope analysis

In Fig. 3 we present the water isotope analysis segment of the CFA system. We follow a slightly modified version of the CFA-water isotope system presented in^14,39^ in which a microflow of liquid water is continuously injected and flash-vaporised in a PID-regulated 170°C oven (T2 in Fig. 3) to which we introduce a stream of dry air (<30 ppmV) with a flow of $\approx 50\,{\rm{cc}}\,{\min }^{-1}$. The liquid microflow is in the order of 2 ${{\rm{\mu lmin}}}^{-1}$ and is achieved by sampling a higher flow of 0.4 ${\rm{ml}}\,{\min }^{-1}$ with a fused silica capillary (ID 50 *μ*m) in a tee-split (T1 in Fig. 3) configuration. The high flow of 0.4 ${\rm{ml}}\,{\min }^{-1}$ is maintained by the peristaltic pump P1 (**Ismatec ISM597D**) that pulls the sample through V1, a 6-port selection valve (**Vici C25-3186EMH**). The latter is used to choose between the CFA sample line or glass containers with water isotope standards or a glass container with background deionised water. We used amber glass 250 ml bottles (**Duran GL45, 250 ml, S/N: 818063604**) with GL-45 thread safety caps (**JR-S-11011**) and inlet solvent stainless steel filters (**IDEX A-302**) with a porosity of 10 *μ*m. Upstream of V1, a parallel configuration of two inline filters (**IDEX A-313**) fitted with stainless steel 20 *μ*m porosity frits (**IDEX A-120**) ensures that the CFA sample stream is free of particles that can deteriorate the smooth operation of the selection valve or the oven capillary. Downstream of the vaporisation oven T2, the vapour sample is introduced into an open split (T3 in Fig. 3) consisting of a stainless steel 1/16 inch tee-split (**Swagelok SS-100-3**), through which the analyzer pulls the necessary flow of $30\,{{\rm{cc\; min}}}^{-1}$.

Our flash-evaporation approach has been shown to yield a vapor sample flow that is not affected by isotopic fractionation issues, thus allowing for a very stable performance with the possibility to also average over longer times in order to achieve very high signal-to-noise ratios. The practically zero-dead volume flow system minimises sample dispersion such that ultra high resolution measurements are possible^39,45^. For optimal results we operate the spectrometer in the [H_2_O] range of 10,000-23,000 ppmV. Tuning of the [H_2_O] level can be achieved with a needle valve installed on the dry air line, which in combination with the open split T3, disturbs neither the sample flow nor the pressure in the optical cavity.

#### The MBS CFA campaign

**Fig. 4:**
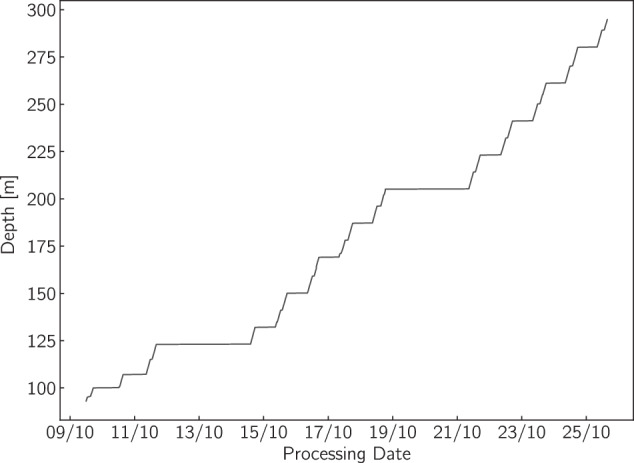
Analysis production during the MBS CFA campaign spanning 13 working days (09/10/2019 - 25/10/2019).

The analysis of the MBS core took place in Copenhagen, at the CFA lab of the Ice and Climate group at the Niels Bohr Institute over 13 working days during October 2019. A total of 195.386 m of ice was processed through the CFA system equivalent to 201.634 m of ice core length when including the pieces of ice removed during the processing of the breaks. Depthwise, the record spans the range 93.082-294.712 m corresponding to the age interval 1736-873 CE^13^. In Fig. 4 we show the processing throughput during the campaign.

#### CFA isotope processing sequence

**Fig. 5:**
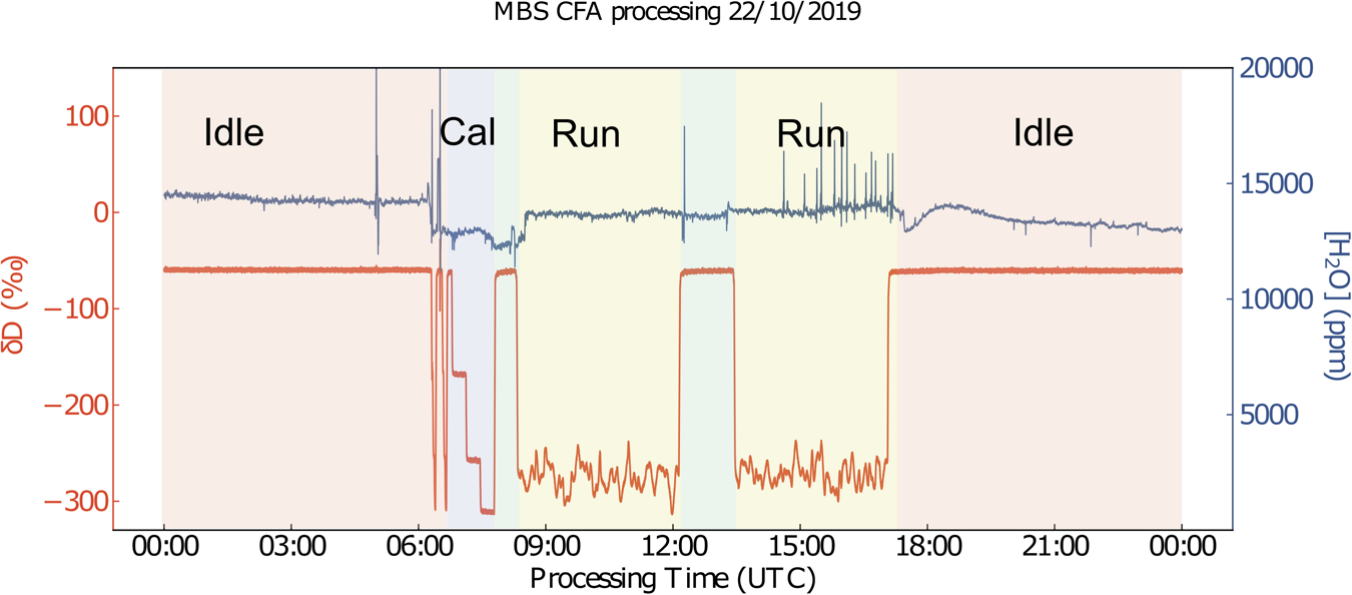
Example of the sequence of events during an analysis day of the MBS CFA campaign. We plot the raw *δ*D signal (left axis) and the water concentration levels in the laser spectrometers optical cavity during a period of 24 hours on the 22^nd^ of October. The pale red, blue and yellow areas of the plot mark the IDLE, VSMOW-SLAP calibration and ice core run phases of the day. The pale green areas correspond to the time when the isotope system was on IDLE mode while the chemical analysis part of the CFA system was being calibrated.

From the water isotope point of view, a typical CFA processing day consists of the Idle, Calibration and Run modes (Fig. 5). In the Idle, mode the selection valve V1 connects to the DI water container feeding the system overnight. These sections were used in order to evaluate the performance of the system with respect to stability, accuracy and long term averaging, by means of Allan variance tests^14,46^ (section Technical Validation).

The processing day starts with a VSMOW-SLAP calibration (section “Cal” colored light blue in Fig. 5) a procedure that involves three local water isotope standards, with the middle one being used as a “check” standard for estimation of the systems accuracy. Each standard water is run for 20 min resulting in a calibration procedure that lasts 60 min in total. The whole sequence can be initiated and monitored remotely via a homemade Python-based valve control and data acquisition script.

When on “Run” mode the system is measuring ice core sample and the selection valve V1 is connected to the “CFA” sample port. As seen in Fig. 5, a processing day contains two sample runs each consisting of 9 ice core bags equivalent to ≈9 m of ice. In the particular example of Fig. 5 from the 22^nd^ of October, the first run (Run #224) consists of the ice core bags 224-232 and the second run (Run #233) consists of ice core bags 233-241. A necessary calibration procedure for the chemical analysis results in a period of Idle mode between the two runs that can last 1-2 h (green areas in Fig. 5).

### Data analysis - Discrete sampling

#### VSMOW-SLAP calibration - Discrete analysis

Three local standards calibrated against the IAEA reference waters SLAP and VSMOW were used to calibrate the raw *δ*^18^O and *δ*D signals to the VSMOW-SLAP scale. All of the in-house standards consist of bulk Antarctic meltwater samples collected from three locations in Antarctica: Dome Summit South (DSS), Lambert Glacial Basin (LGB) and Mount Brown South (MBS). (Table 3). We follow the IAEA recommended procedure^11^ and use the most enriched (DSS) and depleted (LGB) local standards to build a two-point fixed calibration line as:

1 $$\delta {\prime} =\alpha \,\delta +\beta $$

where *δ*’ represents the calibrated signal and the slope *α* and the intercept *β* are given by:

2 $$\alpha =\frac{\delta {{\prime} }_{{\rm{DSS}}}-\delta {{\prime} }_{{\rm{LGB}}}}{{\delta }_{{\rm{DSS}}}-{\delta }_{{\rm{LGB}}}}$$

3 $$\beta =\delta {{\prime} }_{{\rm{DSS}}}-\frac{\delta {{\prime} }_{{\rm{DSS}}}-\delta {{\prime} }_{{\rm{LGB}}}}{{\delta }_{{\rm{DSS}}}-{\delta }_{{\rm{LGB}}}}\,\,{\delta }_{{\rm{DSS}}}$$

The additional “MBS" standard was analysed several times during each run sequence in order to assess the internal accuracy and precision of each analytical run after calibration to the VSMOW-SLAP scale as described above.

### Data analysis - CFA

#### VSMOW-SLAP calibration - CFA analysis

Reporting of the *δ*^18^O and *δ*D signals on the VSMOW-SLAP scale requires a calibration step similar to the one taken for the discrete data and described in section. Following an approach similar to^14,39^ we used three local water isotopic standards calibrated against the primary IAEA reference waters VSMOW and SLAP (Table 4) in order to (i) produce a two fixed-point calibration line defined by the two extreme local standards “–40” and “–22” and (ii) estimate the system’s accuracy by comparing the value of the middle “NEEM” standard post calibration with its nominal value (Table 4). Using the prime notation for the calibrated *δ* signals, raw *δ* measurements are calibrated on the VSMOW-SLAP scale according to the calibration line in Eq. (4)

4 $$\delta {\prime} =\alpha \,\delta +\beta $$

where the slope *α* and the intercept *β* are given by:

5 $$\alpha =\frac{\delta {{\prime} }_{{\rm{-22}}}-\delta {{\prime} }_{{\rm{-40}}}}{{\delta }_{{\rm{-22}}}-{\delta }_{{\rm{-40}}}}$$

6 $$\beta =\delta {{\prime} }_{{\rm{-22}}}-\frac{\delta {{\prime} }_{{\rm{-22}}}-\delta {{\prime} }_{{\rm{-40}}}}{{\delta }_{{\rm{-22}}}-{\delta }_{{\rm{-40}}}}\,\,{\delta }_{{\rm{-22}}}$$

From Eqs. (4)–(6) we calculate the VSMOW calibrated value of the middle “check”standard, which is then compared to the nominal VSMOW value of the “check” standard water (Table 4).

**Table 4:** Copenhagen local standards used for the MBS record VSMOW-SLAP calibrations.

Name	*δ*^18^O	*δ*D
− 22	− 21.86	− 168.32
NEEM	− 33.52	− 257.2
− 40	− 40.02	− 311.1

All values are given in ‰ with respect to VSMOW.

#### Depth assignment

**Fig. 6:**
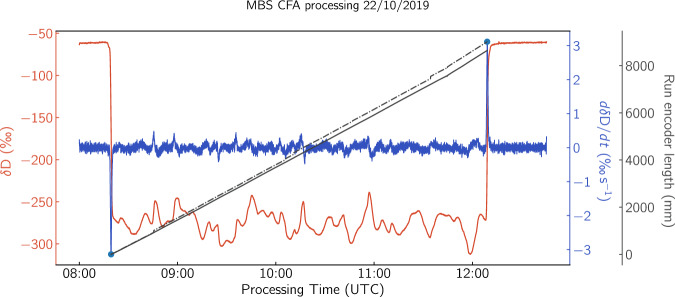
*δ*D of Run 224. The run contains the ice core bags 224-232 and spans ≈9 m. The smoothed derivative of the *δ*D signal (blue curve) is used for the identification of the beginning and the end of the run. With solid black, we plot the run length signal as measured with the wire optical encoder and with the dashed line we show the length signal corrected for the logged internal breaks.

Assigning the depth on each CFA run requires the alignment of the wire depth encoder signal with the *δ*^18^O and *δ*D signals. The **WayCon SX-80** wire encoder delivers a digital signal with a resolution *γ* = 25 countsmm^−1^. The melted length as a function of time can then be calculated by the integration of the differential counts signal $c{\prime} (t)$ as

7 $$l(t)={\int }_{{t}_{i}}^{{t}_{f}}\,{\gamma }^{-1}c{\prime} (t)\,dt$$

where *t*_*i*_ and *t*_*f*_ denote the start and end of the CFA run. Following the detailed logging of the CFA sample rods with respect to internal breaks’ positions and lengths as well as the top depths of every ice core bag it is possible to reconstruct the depth vector *z*(*t*) of each CFA run as a function of elapsed measurement- and Unix Epoch time. In order to transfer the depth scale on the water isotope data, we first locate the beginning and the end of each CFA run in the *δ*D signal. Due to the isotopically heavier value of the background DI water relative to the isotopically light Antarctic sample, CFA run segments are easily distinguishable. We search for the minimum and maximum values of a smoothed version (10-point Bartlett window) of the derivative of the *δ*D signal corresponding to the beginning and end of CFA run respectively. Finally, linear interpolation of the *z*(*t*) signal on the time vector of the isotope signal $\delta {\rm{D}}(t{\prime} )$ sets the isotope measurements on the depth scale of the ice core. In Fig. 6 we show an example of locating the beginning and end of CFA run 224 based on the *δ*D signal and its derivative. We also show the result of the melted length *l*(*t*) calculation based on Eq. (7) without (solid line) and with (dashed-dotted line) the core breaks taken into consideration.

### Data analysis: The age scale

An age scale has been developed for the MBS ice core record, hereafter referred to as MBS2023, resulting in a chronology spanning 1137 a (873–2009 CE)^47^. The construction of the chronology utilises a layer-counting approach, in combination with aligning volcanic horizons by comparing the non-sea sulfate signal (${{\rm{nssSO}}}_{4}^{2-}$) in the MBS, Law Dome^48^, Roosevelt Island Ice Core^49^ as well as the West Antarctic Ice Sheet (WAIS) Divide chronology, WD2014^31^. Annual layers are identified and counted visually in the sea salt and non-sea sulfate signals, the former yielding maxima in austral winter and the latter in austral summer. This type of variability results additionaly in signal peaks for the sulfate to chloride ratio during the austral summer^50^. Austral summer water isotope maxima (*δ*^18^O and *δ*D) are also used in the counting of the annuals. For a detailed description of the dating methods the reader is referred to the dedicated paper by Vance *et al*.^13^.

## Data Records

**Fig. 7:**
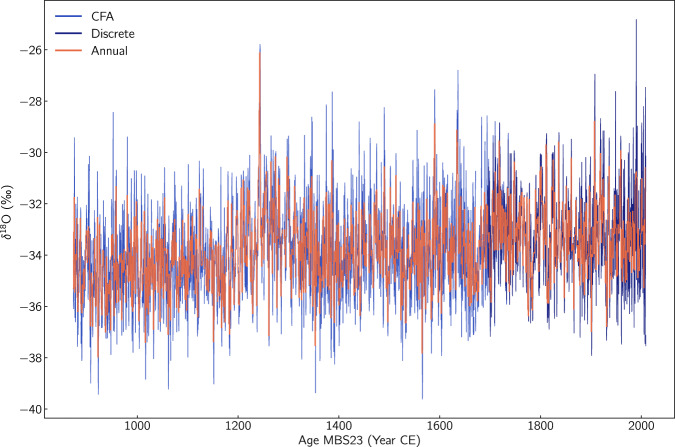
The Mount Brown South water isotope record (*δ*^18^O) as a function of time using the MBS2023 age scale.

The dataset can be found in the Australian Antarctic Data Center (AADC) repository^51^. It consists of two sub-datasets in files **mbs_water_isotopes_a.txt** and **mbs_water_isotopes_b.txt**. The **_a.txt** file contains the discrete data on the 0.03 m resolution spanning the depth range 4-104 m. The temporal resolution of this record varies from ≈ 17 points a^−1^ at the top of the record, to ≈9 points a^−1^ at the bottom of the record. The CFA data can be found in the **_b.txt** file covering a depth range between 93 and 294.7 m. With a depth resolution of 0.005 m this dataset has a temporal resolution of ≈57 points a^−1^ at the top of the record (93 m),while at the bottom of the record (294.7 m) the resolution is ≈45 points a^−1^. We also provide the full record on the MBS2023 timescale only and an annual time resolution. The full record can be found in **mbs_water_isotopes_annual.txt**. In this merged record we use the discrete data from year 2008 CE until year 1695 CE. Thereafter and until the year 873 CE the record is based on the CFA measurements. In Tables 5, 6 and 7 we list and describe the variables included in the data record for the discrete samples and the CFA analysis respectively. The *δ*^18^O signal of the full record is presented on the MBS2023 age scale in Fig. 7

**Table 5:** Name and description of the variables included in the data file mbs_water_isotopes_a containing the discrete samples dataset.

Variable	Unit	Description
Depth_Top	m	Top Depth of data sample
Depth_Mid	m	Middle Depth of data sample
Depth_Bottom	m	Bottom Depth of data sample
MBS2023	a CE	Date based on the MBS_2023_ age scale for the middle depth of the sample
d18	‰	*δ*^18^O of the sample
dD	‰	*δ*D of the sample
d18_precision	‰	1-*σ* of noise for *δ*^18^O estimated from the ”MBS” standard treated as unknown
dD_precision	‰	1-*σ* of noise for *δ*Destimated from the ”MBS” standard treated as unknown
d18_accuracy	‰	Internal accuracy for *δ*^18^O estimated from the ”MBS” standard treated as unknown
dD_accuracy	‰	Internal accuracy for *δ*D estimated from the ”MBS” standard treated as unknown

**Table 6:** Name and description of the variables included in the data file mbs_water_isotopes_b containing the CFA dataset.

Variable	Unit	Description
Depth	m	Depth of data point
MBS2023	a CE	Date based on the MBS_2023_ age scale
d18	‰	*δ*^18^O of the sample
dD	‰	*δ*D of the sample
sigma_d18	‰	1-*σ* of measurement noise for *δ*^18^O calculated from the PSD
sigma_dD	‰	1-*σ* of measurement noise for *δ*D calculated from the PSD

**Table 7:** Name and description of the variables included in the data file mbs_water_isotopes_annual containing the merge of the discrete and the CFA datasets on annual resolution.

Variable	Unit	Description
MBS2023	a CE	Date based on the MBS_2023_ age scale
d18	‰	*δ*^18^O of the 1-y interval
dD	‰	*δ*D of the 1-y interval

## Technical Validation

### Technical Validation: Discrete samples

#### Measurement precision and accuracy

**Fig. 8:**
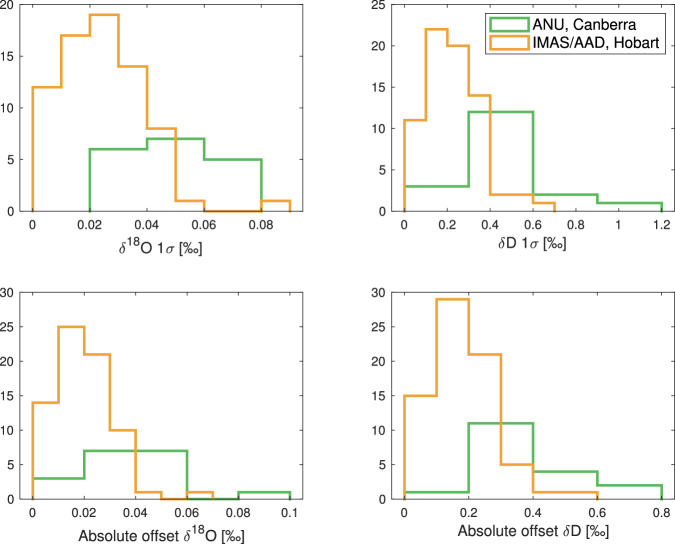
Histograms of the precision (1-*σ*) and mean absolute offset of “check” standards for analytical run at the ANU, Canberra (green, L2140-*i*, *N* = 94) and IMAS/AAD, Hobart (orange, L2130-*i*, *N* = 400).

The measurement accuracy and precision of every run were determined using a “check” standard water, which was treated as an “unknown”. A check standard was run a minimum of three times during each analytical run. Precision was calculated as the standard deviation of the check standards in each run. Accuracy was determined as the mean value of the absolute offset of each calibrated check standard from the assigned value. The mean offsets for *δ*^18^O and *δ*D measured using the L2130-*i* at IMAS were 0.010 ± 0.011 ‰ and 0.181 ± 0.097 ‰ respectively (*N* = 400). For the L2140-*i* at ANU, offsets for *δ*^18^O and *δ*D were 0.040 ± 0.018 ‰ and 0.372 ± 0.150 ‰ respectively (*N* = 94). We present the accuracy and precision metrics in Table 8 and Fig. 8.

**Table 8:** Precision (1-σ) and accuracy of the “check” standard for the L2130-*i* in IMAS (*N* = 400) and the the L2140-*i* in ANU (*N* = 94).

Instrument	*δ*^18^O precision [‰]	*δ*^18^O accuracy [‰]	*δ*D precision [‰]	*δ*D accuracy [‰]
L2130-*i*	0.025 ± 0.014	0.020 ± 0.011	0.230 ± 0.125	0.181 ± 0.097
L2140-*i*	0.049 ± 0.019	0.040 ± 0.018	0.467 ± 0.205	0.372 ± 0.150

### Technical Validation CFA

#### System stability - Allan variance performance

**Fig. 9:**
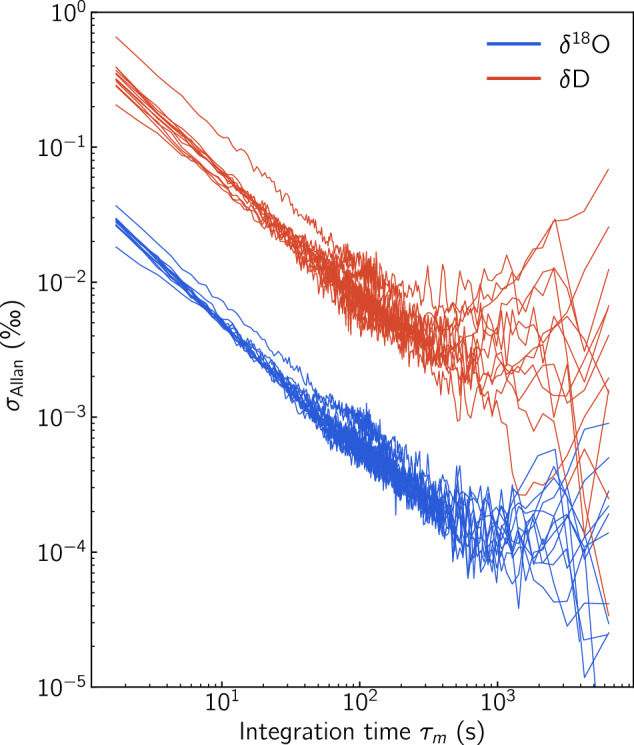
Allan standard deviation as a function of integration time for *δ*^18^O and *δ*D. The metrics are based on the overnight Idle sections in the period 10/10/2019-25/10/2019.

Using the Idle sections of the *δ*^18^O and the *δ*D signals run overnight, we estimated the stability and long term drift of the system using the Allan variance metric^14,36,52^. In this approach a data section with *N* points is “sliced” in *m* subsections with $k=\frac{N}{m}$ number of points, each section being *τ*_*m*_ s long. The Allan variance as a function of the integration time *τ*_*m*_ is then given by the mean value of the squared differences of the adjacent sections’ means:

8 $${\sigma }_{Allan}^{2}\left({\tau }_{m}\right)=\frac{1}{2m}{\sum }_{j=1}^{m}{\left({\bar{\delta }}_{j+1}-{\bar{\delta }}_{j}\right)}^{2}$$

where ${\bar{\delta }}_{j+1}$ and ${\bar{\delta }}_{j}$ are the mean values of neighboring intervals *j* and *j* + 1. The summarised results of the Allan variance tests over 12 days are shown in Fig. 9. Some conclusions can be drawn from the Allan variance tests. Firstly the system’s performance shows a consistent behavior over the 12-days period. Noise is reduced roughly linearly as a function of *τ*_*m*_ with very comparable slopes until the point of an optimum integration time where the *σ*_Allan_ reaches a minimum value, at ≈ 10^3^ s. Beyond that point, *σ*_Allan_ remains below the 10^−3^ and 10^−1^ ‰ level for *δ*^18^O and *δ*D respectively. With an instrument data acquisition rate at ≈ 1 Hz and a reporting resolution of Δ*x* = 5 mm, every data point in the final record is roughly a 5 s average. This integration time corresponds to a *σ*_Allan_ ≈  0.008 ‰ and 0.11 ‰ for *δ*^18^O and *δ*D respectively.

#### VSMOW-SLAP Calibration accuracy

**Fig. 10:**
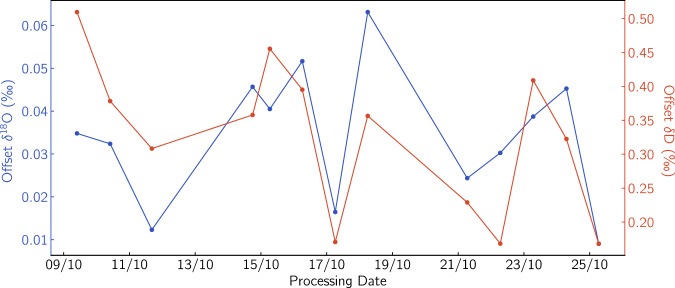
Internal accuracy for the “check” local standard “NEEM” as estimated during the VSMOW-SLAP calibration at the beginning of each analysis day. For *δ*^18^O: 0.034 ± 0.015 ‰, and for *δ*D: 0.32 ± 0.11‰.

As described in section VSMOW-SLAP calibration the internal accuracy of the system can be estimated from the offset between the calibrated and the nominal value of the middle “check” local standard water that is treated as an unknown. The metrics from the calibration accuracy runs are summarised in Fig. 10. The “check” standard offset is below 0.07 and 0.6 ‰ for *δ*^18^O and *δ*D respectively for all 13 VSMOW-SLAP calibration runs during the CFA campaign. The mean value of the offset is 0.034 ± 0.015 ‰ and 0.32 ± 0.11‰ for *δ*^18^O and *δ*D respectively.

#### Power spectral density noise estimation

**Fig. 11:**
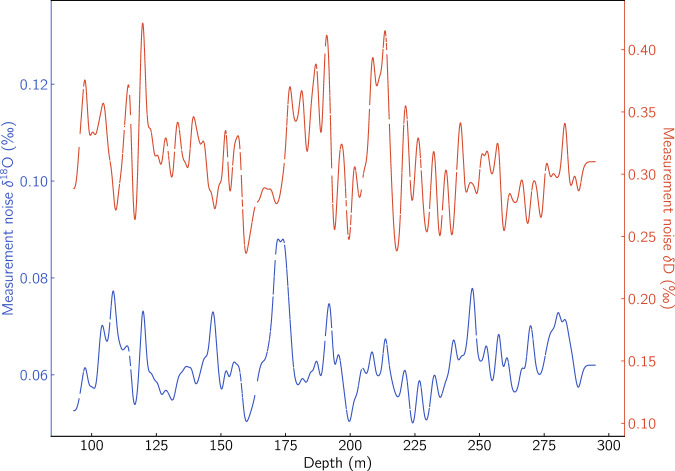
Measurement noise of the *δ*^18^O and *δ*D signals as a function of ice core depth estimated from the noise component of the power spectral density of the isotope signals.

**Fig. 12:**
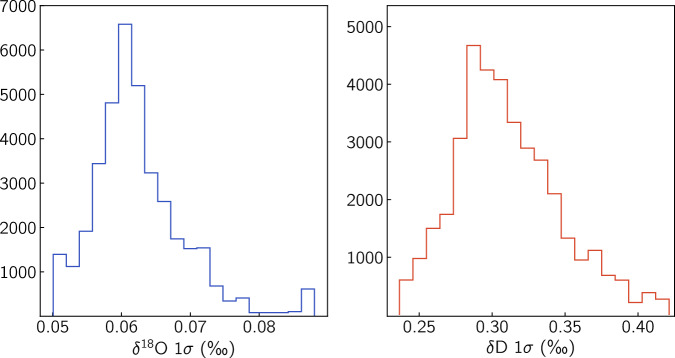
Histograms of the CFA measurement noise in Fig. 11. *δ*^18^O: 0.063 ± 0.008 ‰, *δ*D: 0.311 ± 0.036‰.

While the Allan variance and the VSMOW-SLAP accuracy checks are very useful tools in assessing the performance of the system, they cannot account for noise occurring during the measurement of the ice core sample. This noise could be due to instabilities in the liquid sample flow or the melting of the ice core and typically result in the inclusion of air bubbles in the system. These can in turn disturb the continuous vaporisation process yielding a fluctuating [H_2_O] concentration in the spectrometer’s optical cavity. The large number of data points in the record due to the high resolution, allows for a more pragmatic and direct estimation of the records’ noise levels. Similar to the approach followed in^39^, the noise of a data section can be deduced from the noise component of the power spectral density (PSD) of the *δ*^18^O, *δ*D signals by calculating the integral:

9 $${\sigma }^{2}={\int }_{0}^{{f}_{c}}| \widehat{\eta }(f){| }^{2}df$$

where *f*_*c*_ = 1/2Δ*x* = 100 m^−1^ is the Nyquist frequency. With $\widehat{\delta }(f)$ being the Fourier transform of the *δ*^18^O,*δ*D signals, $| \widehat{\eta }(f){| }^{2}$ can be estimated by a linear fit on the flat high-frequency part of the power spectral density $| \widehat{\delta }(f){| }^{2}$. For this study, we implement a rolling window with a size of 1000 points and calculate the Discrete Fast Fourier Transform (DFFT) for real-valued data^53,54^ from which an estimate of the PSD $| \widehat{\delta }(f){| }^{2}$ can be obtained. The spectra reveal an easy to identify, white-noise plateau the level of which we obtain by means of a least squares fit of a zero-order polynomial for frequencies higher than 50 m^−1^. Subsequently we filter the measurement noise signals with a 200 point Gaussian filter. The result of the calculation is given in Fig. 11 revealing a noise level of 0.063 ± 0.008 ‰ and 0.314 ± 0.04‰ for *δ*^18^O and *δ*D respectively. Histograms of the measurement noise distributions are also calculated and illustrated in Fig. 12.

#### Discrete - CFA comparison

**Fig. 13:**
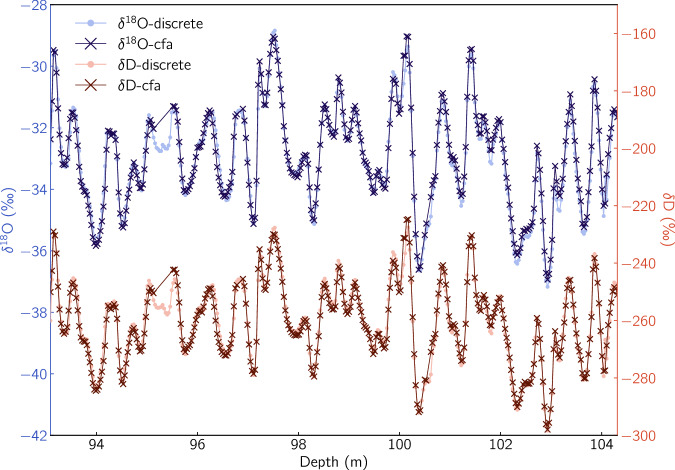
A visual comparison between the discrete and the CFA datasets for *δ*^18^O and *δ*D. The CFA data are averaged on the resolution of the discrete sampling.

**Fig. 14:**
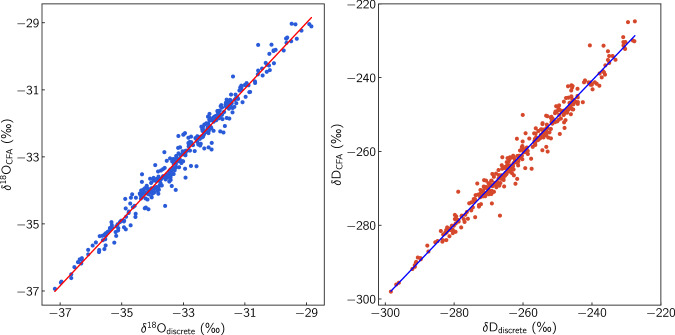
Correlation between the discrete and CFA averaged datasets for *δ*^18^O and *δ*D. The correlation concerns the depth range 93-104 in which both techniques were used for the isotope analysis and is equal to 0.989 and 0.988 for *δ*^18^O and *δ*D respectively.

The overlap between the two records in the depth range 93-104 m allows for a comparison between the two datasets. Such a comparison is useful in identifying non systematic errors that typically cannot be characterised using the validation procedures described in the previous sections. Firstly, we average the CFA dataset on the resolution of the discrete samples. The comparison plot overlaying the CFA measurements on the discrete data shows a very good agreement between the time series (Fig. 13) revealing the ice core isotope signal for both *δ*^18^O and *δ*D without any outliers or obvious discrepancies. A step further, we plot the signals against each other (Fig. 14) in order to investigate further for possible outliers on both signals. The correlation coefficients derived from this comparisons are 0.989 and 0.988 for *δ*^18^O and *δ*D respectively. The plots reveal a number of data points with a relatively high deviation from the correlation line thus showing indication for a non-normal distribution of the measurement errors.

**Fig. 15:**
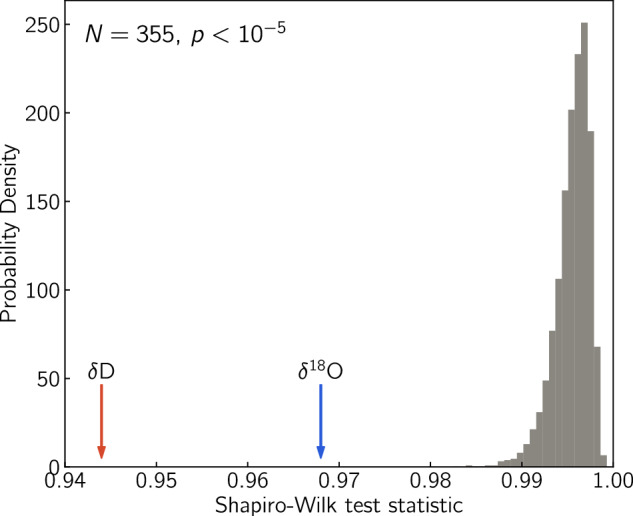
Shapiro-Wilk normality test for the residuals between the CFA and discrete data for *δ*^18^O and *δ*D datasets for the depth range 93-104 m. The red and blue arrow signify the value of the Shapiro-Wilk statistic for the *δ*D and *δ*^18^O residuals respectively. The gray distribution demonstrates the results of the Monte Carlo simulation using 10000 realisations of a series with N = 355 drawn from a normal distribution. The p-value for both the *δ*D and the *δ*^18^O residuals is <10^−5^ signifying that it is very unlikely that the residuals between the discrete and the CFA data series are normally distributed.

In order to investigate this we form a hypothesis test using the Shapiro-Wilk statistic^55^ for departure from normality. In particular, we use a modified version^54,56^ of the algorithm introduced by Royston^57^, that is optimised for larger datasets with size 3 ≤ *n* ≤ 5000. We test the null-hypothesis that the residuals between the two series are distributed normally, by comparing the Shapiro statistic of the data residuals to the statistic of 10,000 realisations of randomly distributed residuals following a Monte Carlo approach. The Shapiro-Wilk statistic yields values closer to 1 for normally distributed variables. The Monte Carlo simulation yields a Shapiro-Wilk statistic equal to 0.969 and 0.944 for the residuals of the *δ*^18^O and the *δ*D series respectively (Fig. 15). The p-value for both estimates is below 10^−5^. This is a very strong indication that the null-hypothesis is false and thus the residuals between the CFA and discrete data are not normally distributed for either of the isotope ratios measured. This result indicates that despite the sound validation procedures for accuracy and precision as well as depth registration, non-systematic sources of error can still exist and go unnoticed. In practical terms, the signal to noise ratio in the isotope signal is so high that the observed discrepancies between the discrete and the CFA data are insignificant.

## Data Availability

The Python code used for this study is available on GitHub (https://github.com/vgkinis/mbs_cfa_isotopes).
